# Exosomes from high glucose-treated glomerular endothelial cells trigger the epithelial-mesenchymal transition and dysfunction of podocytes

**DOI:** 10.1038/s41598-017-09907-6

**Published:** 2017-08-24

**Authors:** Xiaoming Wu, Yanbin Gao, Liping Xu, Wanyu Dang, Huimin Yan, Dawei Zou, Zhiyao Zhu, Liangtao Luo, Nianxiu Tian, Xiaolei Wang, Yu Tong, Zheji Han

**Affiliations:** 1 Beijing Key Lab of TCM Collateral Disease Theory Research, School of Traditional Chinese Medicine, Capital Medical University, No.10, Youanmenwai, Xitoutiao, Fengtai District, Beijing, 100069 China; 2 Beijing Children’s Hospital, Capital Medical University, NO. 56, Nanlishi Road, Xi Cheng District, Beijing, 100045 China

## Abstract

New data indicate that abnormal glomerular endothelial cell (GEC)-podocyte crosstalk plays a critical role in diabetic nephropathy (DN). The aim of our study is to investigate the role of exosomes from high glucose (HG)-treated GECs in the epithelial-mesenchymal transition (EMT) and dysfunction of podocytes. In this study, exosomes were extracted from GEC culture supernatants and podocytes were incubated with the GEC-derived exosomes. Here, we demonstrate that HG induces the endothelial-mesenchymal transition (EndoMT) of GECs and HG-treated cells undergoing the EndoMT secrete more exosomes than normal glucose (NG)-treated GECs. We show that GEC-derived exosomes can be internalized by podocytes and exosomes from HG-treated cells undergoing an EndoMT-like process can trigger the podocyte EMT and barrier dysfunction. Our study reveals that TGF-β1 mRNA is enriched in exosomes from HG-treated GECs and probably mediates the EMT and dysfunction of podocytes. In addition, our experimental results illustrate that canonical Wnt/β-catenin signaling is involved in the exosome-induced podocyte EMT. Our findings suggest the importance of paracrine communication via exosomes between cells undergoing the EndoMT and podocytes for renal fibrosis in DN. Thus, protecting GECs from the EndoMT and inhibiting TGF-β1-containing exosomes release from GECs is necessary to manage renal fibrosis in DN.

## Introduction

Diabetic nephropathy (DN), a severe microvascular complication of diabetes, is the leading cause of end-stage renal disease (ESRD) worldwide^1^. DN is clinically characterized by proteinuria, which is the manifestation of damage to the glomerular filtration barrier^2^. The glomerular filtration barrier consists of three layers: the fenestrated endothelial layer, the glomerular basement membrane (GBM), and the layer of visceral epithelial cells called podocytes, which reside in the GBM outside the glomerular capillaries and possess foot processes connected by the slit diaphragm^3^. Podocytes act as the final barrier to macromolecular flow into the urinary filtrate and are integral to the maintenance of the glomerular filtration barrier. However, as a terminally differentiated cell, podocytes have minimal capacity to self-replicate and are extremely vulnerable to cellular injury^4^. Podocytes dysfunction and depletion play a fundamental role in the onset and progression of proteinuria and glomerulosclerosis in DN^5^. Emerging evidence indicates that the epithelial-mesenchymal transition (EMT) of podocytes after injury is a mechanism underlying podocyte dysfunction and podocytopenia in DN^6^. In response to injurious stimuli, podocytes can undergo a phenotypic switch characterized by the loss of expression of highly specialized podocyte markers such as nephrin, P-cadherin, zonula occludens-1 (ZO-1) and Wilms’ tumor 1 (WT 1), while acquiring the expression of new mesenchymal markers such as fibroblast-specific protein-1 (FSP-1), desmin, α-Smooth Muscle Actin (α-SMA), matrix metalloproteinase-9 (MMP-9), type I collagen (Col-I), type IV collagen (Col-IV) and fibronectin (FN)^7–9^. Transforming growth factor-β1 (TGF-β1), a potent profibrotic cytokine, is significantly up-regulated in the kidneys of DN patients and is a potent trigger of the podocyte EMT^10^. Canonical Wnt/β-catenin signaling mediates TGF-β1-triggered podocyte injury and proteinuria^11^.

A wealth of data indicates that intercellular communication occurs between resident cells within the glomerulus^12, 13^. Structurally, podocytes line the urinary space and are separated from glomerular endothelial cells (GECs) by the GBM. GEC-podocyte crosstalk has recently been recognized to play a critical role in maintaining the integrity of the glomerular filtration barrier, and alternative GEC-derived secreted factors may profoundly influence the function of podocytes under pathological conditions^14^. For example, an endothelial nitric oxide synthase (eNOS) deficiency contributes to podocytes injury, leading to leakage of albumin into the urine of diabetic patients^15^. Soluble mediators secreted from GECs under chronic laminar shear stress impair podocyte barrier resistance^16^. Endothelin-1 released by podocytes leads to oxidative stress in endothelial mitochondrial, which in turn increases podocyte apoptosis in progressive glomerulosclerosis^17^. In summary, accumulated evidence demonstrates that GEC dysfunction, one of the earliest events in diabetes, may cause podocyte damage by releasing paracrine mediators, and the identification of novel mediators of GEC-podocyte communication may lead to the development of more effective strategies to treat DN^12, 14^.

The release of exosomes, membrane-bound vesicles 30–100 nm in size that are secreted into the extracellular space by various cell types, has emerged as a novel and significant mechanism of intercellular communication in recent years^18^. Exosome-mediated deliveries of mRNA, microRNA, proteins and signaling molecules play important roles in cell-to-cell communication^19^. Recent sudies indicate that endothelial cells can communicate with target cells or the surrounding environment through the release of exosomes. For example, exosomes derived from hypoxic endothelial cells can increase collagen crosslinking activity via up-regulation of lysyl oxidase-like 2^20^. Endothelial exosomes can inhibit breast cancer cell proliferation and invasion via the transfer of microRNA-503 in response to chemotherapy treatment^21^. Exosomes derived from cardiac endothelial cells can induce B cells to release TGF-β, which suppresses the effector T cell proliferation^22^. Existing studies show that some cell-derived exosomes have the capacity to induce differentiation or transdifferentiation of target cells through the delivery of TGF-β under pathological conditions. For example, exosomes released from gastric cancer cells can trigger the differentiation of umbilical cord-derived mesenchymal stem cells into carcinoma-associated fibroblasts via the transfer of TGF-β^23^. Lung cancer cell-derived exosomes can regulate the tumor cells migration by transferring TGF-β^24^. In cancer, the exosomal delivery of TGF-β is capable of driving fibroblast-to-myofibroblast differentiation^25^. In our previous studies, we revealed that exosomes containing TGF-β1 secreted by high glucose (HG)-treated GECs could activate mesangial cells to promote renal fibrosis^26^. However, in diabetes, whether GEC-derived exosomes can fuse with podocytes and trigger the EMT of podocytes is not clear.

Collectively, an abnormal interaction between GECs and podocytes is believed to play a critical role in the pathogenesis of DN. In this study, we examined whether exosomes released by HG-treated GECs could transmit information to podocytes and induce the EMT and dysfunction of podocytes.

## Results

### HG induces an EndoMT-like process in cultured GECs

**Figure 1:**
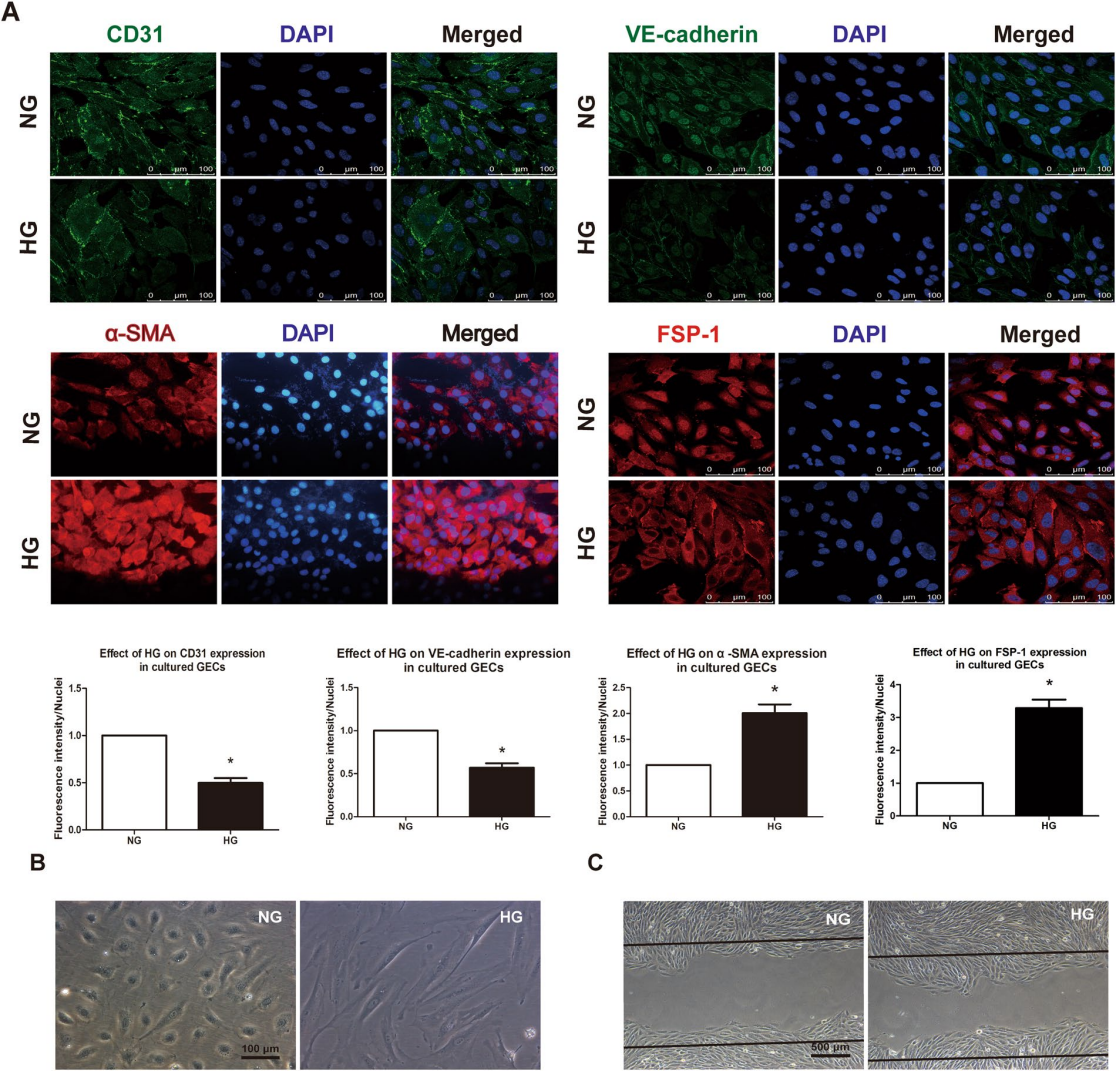
HG induces an EndoMT-like process in cultured GECs. (**A**) Compared to expression in the NG group, HG markedly downregulated the expression of endothelial markers CD31 and VE- cadherin and up-regulated the expression of the mesenchymal markers α-SMA and FSP-1 in cultured GECs. The results are presented as the mean ± SD. *p < 0.05 versus NG group (Student’s *t*-test; *n* = 6). (**B**) HG induced a significant change in cell morphology, from cobblestone like cells to long spindle shaped cells, which is characteristic of cells undergoing the EndoMT. (*n* = 3 for each group) (**C**) Compared to migration in the NG group, HG significantly enhanced the migration capacity of GECs (24 h). (*n* = 3 for each group). NG group, normal glucose group; HG group, high glucose group.

Previous studies have demonstrated that the endothelial-to-mesenchymal transition (EndoMT) contributes to the early development of diabetic renal interstitial fibrosis and glomerulosclerosis^27, 28^. During the EndoMT, endothelial cells lose their endothelial markers, such as CD31 and VE-cadherin, while acquiring mesenchymal markers, such as α-SMA and FSP-1. In our *in vitro* experiment, GECs were cultured in normal glucose (NG; 5.6 mmol/L glucose +24.5 mmol/L mannitol) or HG conditions (30 mmol/L glucose) for 24 h to examine whether HG induces an EndoMT-like process in GECs. As demonstrated in Fig. 1A, HG markedly reduced the expression of the endothelial markers CD31 and VE-cadherin and significantly increased the expression of the fibroblast-like markers α-SMA and FSP-1 in GECs. GECs cultured in the HG environment changed dramatically from cells with a typical cobblestone morphology to elongated spindle-shaped cells (Fig. 1B). In addition, as shown in Fig. 1C, HG obviously increased the migration capacity of GECs (24 h). These results confirm that HG can induce an EndoMT-like process in GECs.

### HG-treated cells undergoing the EndoMT secrete more exosomes than NG-treated GECs

**Figure 2:**
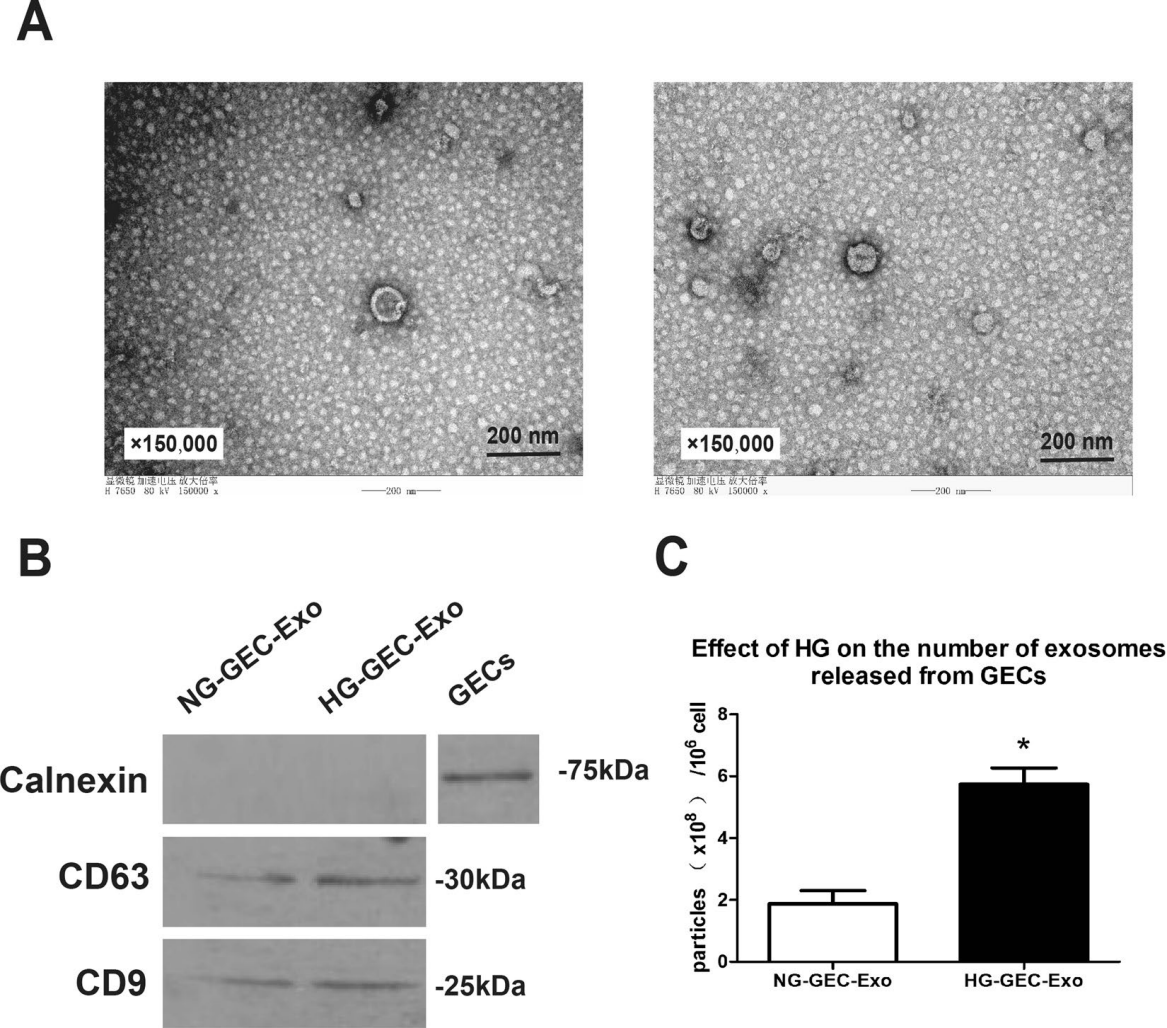
HG-treated cells undergoing the EndoMT secrete more exosomes than NG-treated GECs. (**A**) Exosomes extracted from GECs were observed by TEM. Magnification: ×150,000. (*n* = 4) (**B**) Protein blots of exosomes derived from the same number of NG- and HG-treated GECs using antibodies for calnexin, CD63 and CD9. In addition, the expression of calnexin in GECs was also examined by Western blotting. (*n* = 3 for each index) (**C**) Quantification of exosomes isolated from NG- and HG-treated GECs normalized by cell number. Compared to the NG-GEC-Exo group, HG-treated cells undergoing the EndoMT released a higher number of exosomes. The results are presented as the mean ± SD. *p < 0.05 versus NG-GEC-Exo group (Student’s *t*-test; *n* = 6). NG-GEC-Exo group, exosomes derived from NG-treated GECs; HG-GEC-Exo group, exosomes derived from HG-treated GECs.

Exosomes were isolated from the cell-culture supernatants of the same number of NG-treated GECs and HG-treated GECs and then observed by transmission electron microscopy (TEM) and detected by Western blotting. TEM analysis showed that GEC-derived exosomes were round, membrane-bound vesicles with a diameter of 30–100 nm (Fig. 2A). Western blotting analysis revealed that these GEC-derived exosomes were highly positive for the exosomal marker proteins, such as CD63 and CD9, and were negative for calnexin, which is a marker of the endoplasmic reticulum and often associated with cellular debris, suggesting that our exosome preparations were not contaminated with cells (Fig. 2B). In addition, the expression of exosomal marker proteins was higher in exosome preparations from HG-treated cells undergoing the EndoMT than in NG-treated GECs, indicating that cells undergoing the EndoMT might release more exosomes (Fig. 2B). To further confirm whether cells undergoing the EndoMT secrete more exosomes, we quantified exosome secretion using a EXOCET Exosomes Quantitation Kit and a FluoroCet Exosome Quantitation Kit (System Biosciences, USA). The data show that cells undergoing the EndoMT produced an increased number of exosomes compared to normal GECs.

Taken together, these results indicate that GEC-derived exosomes can be successfully purified and HG-treated cells undergoing the EndoMT release more exosomes than NG-treated cells.

### GEC-derived exosomes are internalized by podocytes

**Figure 3:**
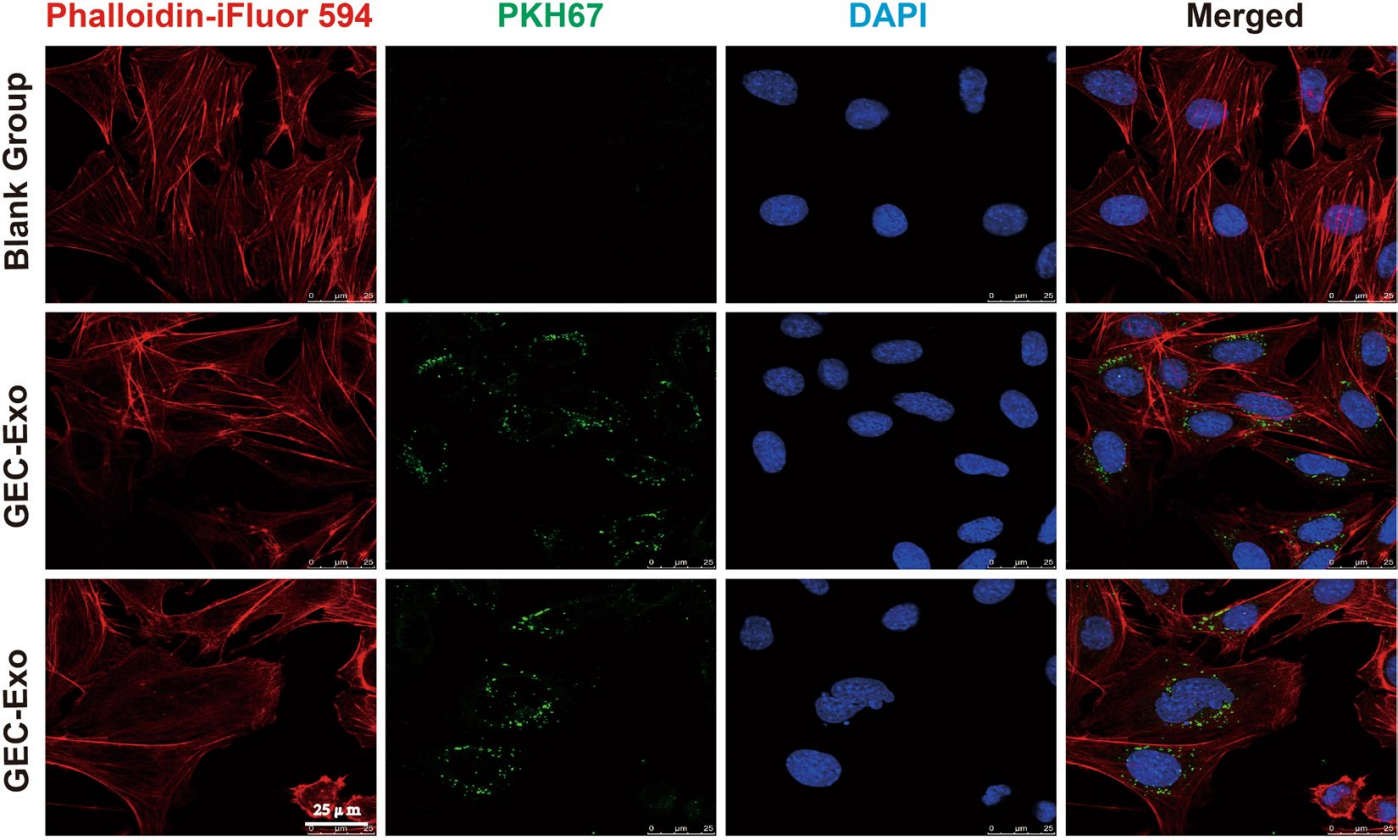
GEC-derived exosomes are internalized by podocytes. Exosomes extracted from GEC culture supernatants were labeled with lipophilic fluorescent dye PKH67 and co-cultured with podocytes for 24 h. Subsequently, the podocytes were stained with phalloidin-iFluor 594 conjugate to display the F-actin distribution. Blank group, podocytes without exosome co-incubation; GEC-Exo group, podocytes incubated with GEC-derived exosomes. Independent experiments were repeated 3 times.

Exosomes were labeled with the green lipophilic fluorescent dye PKH67 and co-cultured with podocytes for 24 h to investigate whether GEC-derived exosomes could be internalized by podocytes. Then, the podocytes were stained with fluorescent phalloidin for the intracellular staining of the F-actin cytoskeleton. Confocal laser microscopy showed that PKH67-labeled exosomes were localized to the perinuclear region of podocytes (Fig. 3), indicating that GEC-derived exosomes can be internalized by podocytes.

### Exosomes from HG-treated cells undergoing an EndoMT-like process trigger the podocyte EMT and barrier dysfunction

**Figure 4:**
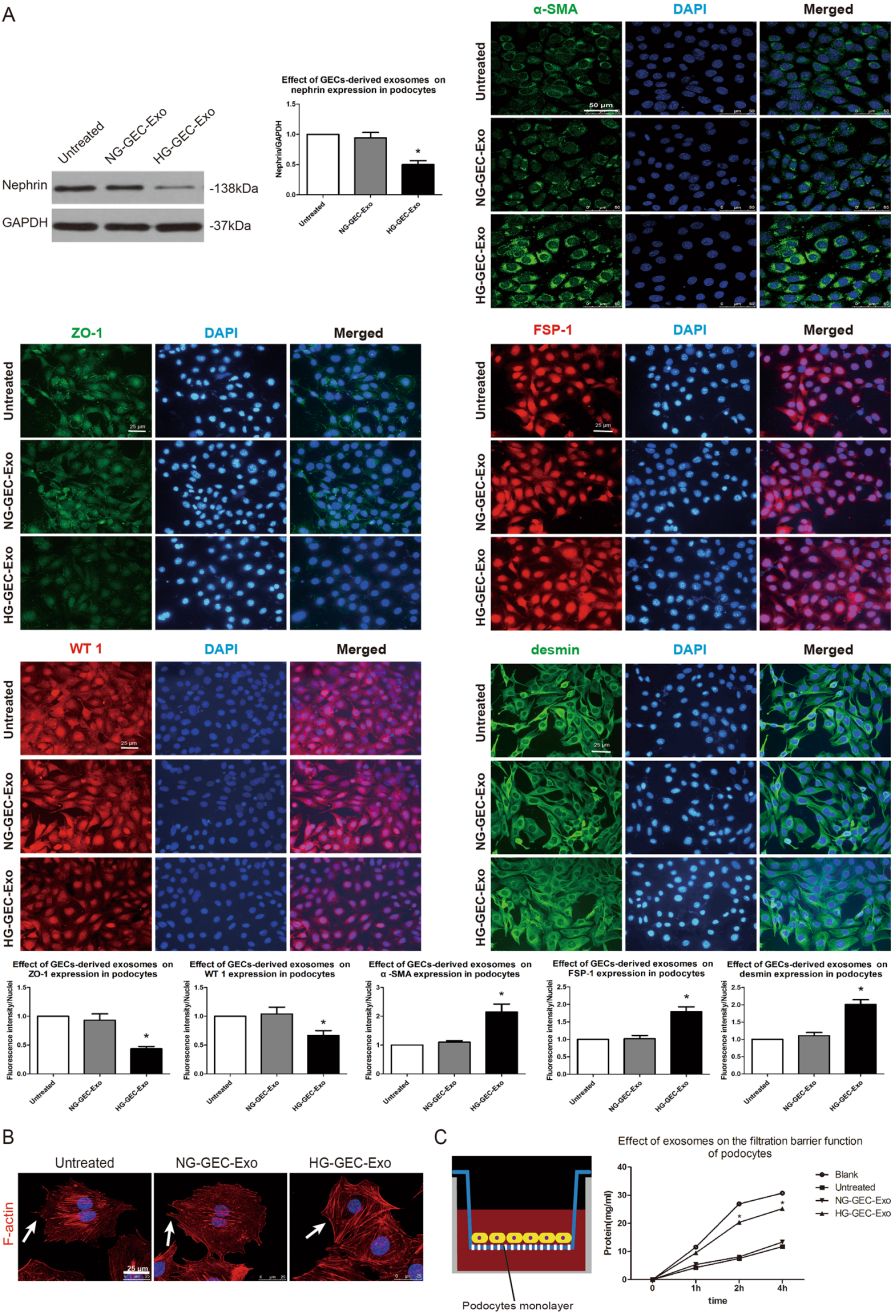
Exosomes from HG-treated cells that underwent an EndoMT-like process trigger the podocyte EMT and barrier dysfunction. (**A**) Western blotting and immunofluorescence staining show that exosomes from HG-treated cells undergoing the EndoMT significantly suppressed nephrin, ZO-1, and WT 1 expression and induced α-SMA, FSP-1, and desmin expression in podocytes. The results are presented as the mean ± SD. *p < 0.05 (ANOVA; *n* = 4; significantly different from the Untreated group and NG-GEC-Exo group). (**B**) Exosomes from HG-treated cells undergoing the EndoMT induced cytoskeletal disorganization and foot process effacement in cultured podocytes. Independent experiments were repeated 3 times. (**C**) Podocytes cultured in the upper chamber were incubated with or without exosomes for 24 h, and the BSA permeability of podocyte monolayers was then examined. The results show that the filtration barrier function of podocytes was severely damaged after the EMT triggered by exosomes from HG-treated cells undergoing the EndoMT. *p < 0.05 (ANOVA; *n* = 3; significantly different from the Untreated group and NG-GEC-Exo group). Blank group, permeable supports in the upper chamber without cultured podocytes; Untreated group, podocytes without exosome treatment; NG-GEC-Exo group, podocytes incubated with exosomes isolated from NG-treated GECs; HG-GEC-Exo group, podocytes incubated with exosomes isolated from HG-treated GECs.

The loss of epithelial markers (nephrin, ZO-1, WT 1) and gain of mesenchymal markers (α-SMA, desmin, FSP-1) are key hallmarks of the podocyte EMT^10^. To investigate the effect of exosomes on podocyte phenotype, the expression levels of nephrin, ZO-1, WT 1, α-SMA, desmin, and FSP-1 protein were examined by Western blotting and immunofluorescence. The results revealed that nephrin, ZO-1, WT 1 expression were remarkably decreased and that α-SMA, desmin, and FSP-1 expression was significantly increased in podocytes treated with exosomes from HG-treated cells undergoing an EndoMT-like process but not in untreated podocytes or podocytes co-cultured with exosomes from NG-treated GECs (Fig. 4A).

Dedifferentiated podocytes exhibit significant cytoskeletal disorganization, which leads to podocyte foot-process effacement^29^. F-actin in podocytes was labeled with phalloidin to determine the effect of exosomes on podocyte morphology and the cytoskeleton. Confocal images show that F-actin was highly organized and the specialized foot process architecture was clear in untreated podocytes and podocytes co-cultured with exosomes from NG-treated GECs, whereas the podocytes incubated with exosomes from cells that underwent an EndoMT-like process displayed significant F-actin disorganization and foot process effacement (Fig. 4B).

Nephrin and ZO-1 are important components of the slit diaphragm. Therefore, defects in their expression would certainly damage the integrity of the slit diaphragm and impair the filtration barrier function of the podocyte monolayer, leading to proteinuria and glomerulosclerosis^30^. An albumin influx assay was used to measure bovine serum albumin (BSA) diffusion across the podocyte monolayer to examine the effect of exosomes on the filtration barrier function of podocytes^8, 31^. As depicted in Fig. 4C, differentiated podocytes were co-cultured with exosomes from NG- or HG- treated GECs for 24 h and then subjected to the albumin influx assay. The diffusion of BSA across membrane filters onto which podocytes were incubated with exosomes from NG-treated GECs was not different from that across membrane filters onto which untreated podocytes were cultured. In contrast, BSA diffusion was markedly higher across membrane filters that contained podocytes co-cultured with exosomes from HG-treated GECs.

### Exosomes from cells undergoing the EndoMT increase TGF-β1 expression and activate Wnt/β-catenin signaling in podocytes

**Figure 5:**
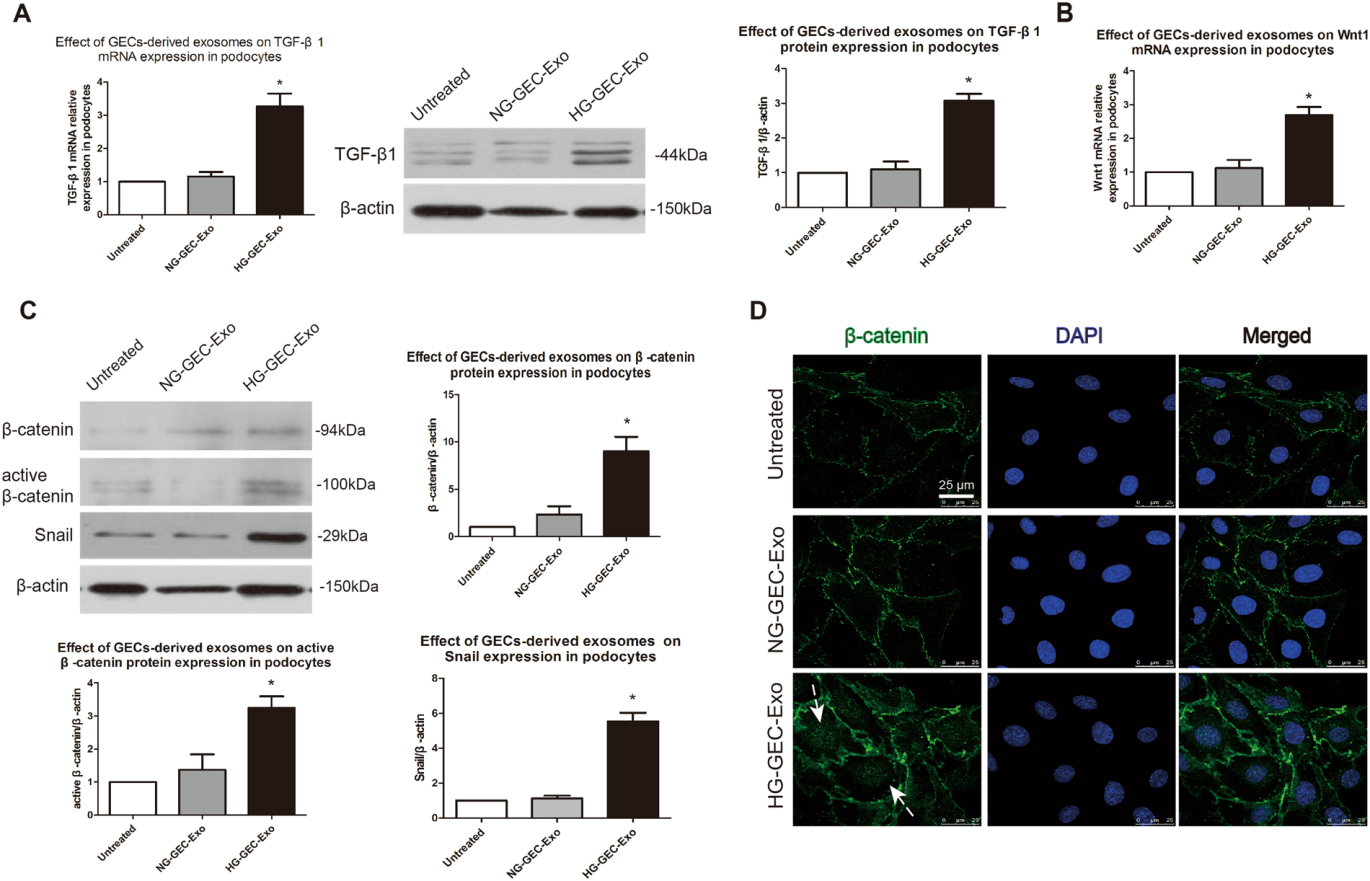
Exosomes from cells undergoing the EndoMT increase TGF-β1 expression and activate Wnt/β-catenin signaling in podocytes. (**A**) RT-PCR and Western blot analysis indicated that exosomes from HG-treated GECs increased TGF-β1 expression in cultured podocytes. The results are presented as the mean ± SD. *p < 0.05 (ANOVA; *n* = 3; significantly different from the Untreated group and NG-GEC-Exo group). (**B**) RT-PCR demonstrated an increased expression of Wnt1 mRNA in cultured podocytes after treatment with exosomes from HG-treated GECs. *p < 0.05 (ANOVA; *n* = 4; significantly different from the Untreated group and NG-GEC-Exo group). (**C**, **D**) Western blot analysis and immunofluorescence staining showed that exosomes from HG-treated GECs increased total β-catenin, active β-catenin and Snail protein expression and promoted β-catenin nuclear translocation in podocytes. The results are presented as the mean ± SD. *p < 0.05 (ANOVA; *n* = 3; significantly different from the Untreated group and NG-GEC-Exo group).

TGF-β1 is a strong inducer of the EMT of podocytes, and the canonical Wnt/β-catenin signaling pathway mediates TGF-β1-triggered EMT and dysfunction in podocytes^7, 11^. To determine whether TGF-β1 and the Wnt/β-catenin signaling pathway are involved in the exosome-induced podocyte EMT, we examined the expression of TGF-β1, Wnt1, β-catenin, and Snail, one of the target genes of Wnt/β-catenin, in podocytes by RT-PCR, Western blotting and immunofluorescence staining. Figure 5A shows that administration of exosomes from cells undergoing an EndoMT-like process markedly increased TGF-β1 mRNA and protein levels in podocytes compared to the effect of exosomes from NG-treated GECs. RT-PCR results show a significant induction of Wnt1 mRNA expression in podocytes by exosomes from cells undergoing the EndoMT compared to the effect of exosomes from NG-treated GECs (Fig. 5B). Western blotting analysis indicated that, compared to exosomes from NG-treated GECs, total β-catenin, active β-catenin and Snail levels were markedly increased in podocytes after treatment with exosomes from cells undergoing an EndoMT-like process (Fig. 5C). In addition, immunofluorescence staining show that, compared to treatment with exosomes from NG-treated GECs, treatment with exosomes derived from cells undergoing the EndoMT up-regulated the expression of β-catenin and clearly caused β-catenin to undergo nuclear translocation, indicating the activation of canonical Wnt/β-catenin signaling (Fig. 5D).

### Inhibition of exosomal TGF-β1 preserves podocyte phenotypes

**Figure 6:**
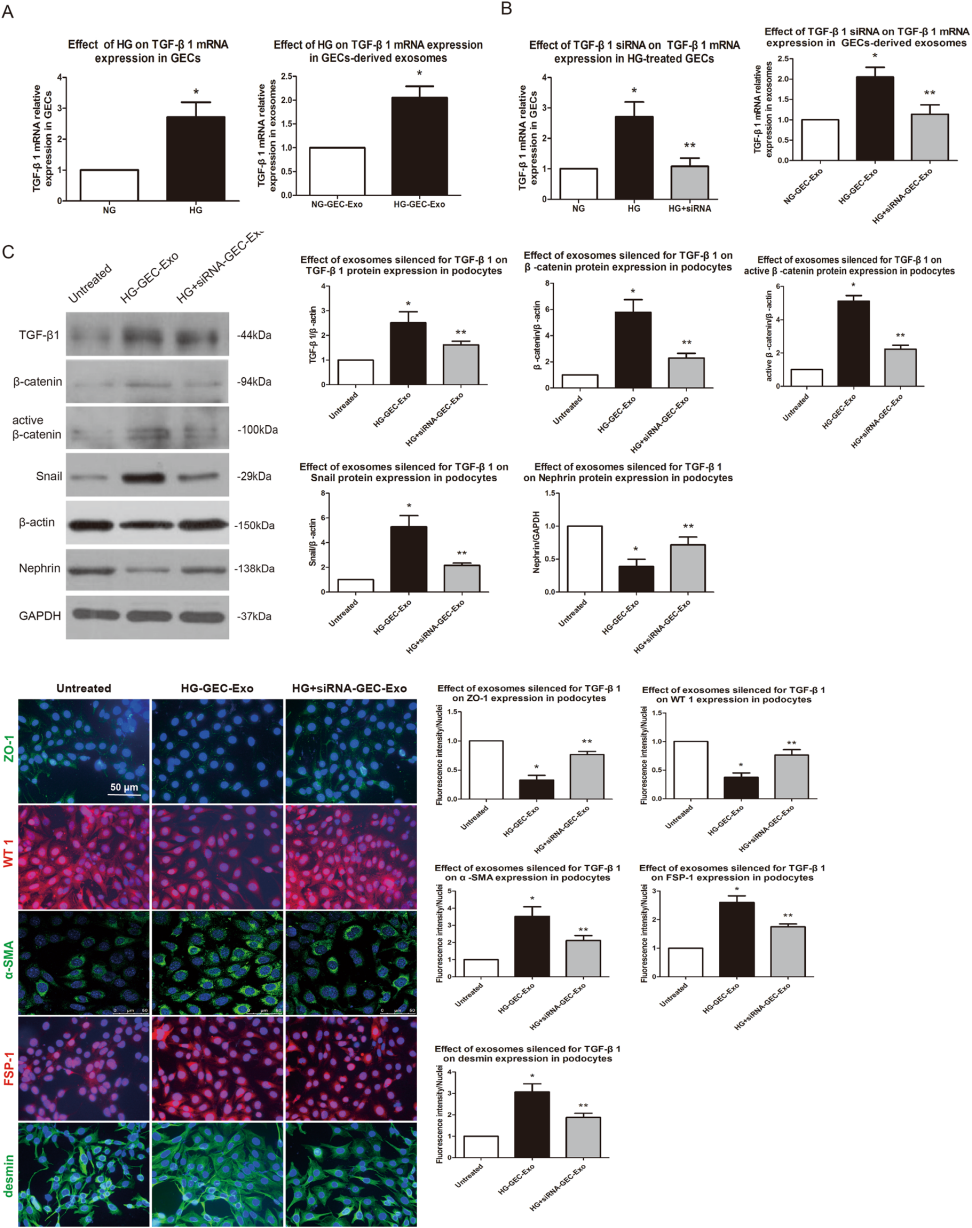
Inhibition of exosomal TGF-β1 preserves podocyte phenotypes. (**A**) HG increased TGF-β1 mRNA expression in both GECs and related exosomes. The results are presented as the mean ± SD. *p < 0.05 (Student’s *t*-test; *n* = 3; significantly different from the NG group and NG-GEC-Exo group). (**B**) A remarkable decrease in TGF-β1 mRNA expression was observed in HG-treated GECs incubated with TGF-β1 siRNA and related exosomes. The results are expressed as the mean ± SD. *p < 0.05 (ANOVA; *n* = 3; significantly different from the NG group and NG-GEC-Exo group). **p < 0.05 (ANOVA; *n* = 3; significantly different from the HG group and HG-GEC-Exo group). (**C**) Compared to expression in the HG-GEC-Exo group, TGF-β1, β-catenin, active β-catenin, Snail, α-SMA, FSP-1, and desmin expression decreased and nephrin, ZO-1, and WT1 expression increased markedly in the podocytes incubated with exosomes silenced for TGF-β1. The results are presented as the mean ± SD. *p < 0.05 (ANOVA; *n* = 3; significantly different from the Untreated group). **p < 0.05 (ANOVA; *n* = 3; significantly different from the HG-GEC-Exo group).

Exosomes play critical roles in intercellular communication through the horizontal transfer of proteins, RNAs and lipids to target cells. Therefore, we hypothesized that exosomal TGF-β1 might be responsible for the increased expression of TGF-β1 in exosome-treated podocytes. We examined TGF-β1 mRNA expression in GECs and related exosomes. As shown in Fig. 6A, compared to expression in NG-treated GECs, TGF-β1 mRNA expression was higher in HG-treated cells undergoing an EndoMT-like process, and TGF-β1 levels were higher in exosomes derived from these cells, suggesting that exosomal TGF-β1 mRNA may contribute to the podocyte EMT.

To investigate whether the exosomal TGF-β1 mRNA is functionally important for the podocyte EMT, TGF-β1 small interfering RNA (siRNA) was used to silence TGF-β1 mRNA in HG-treated GECs. Then we assessed the expression of TGF-β1 mRNA in HG-treated GECs and related exosomes. RT-PCR results show that transient transfection with TGF-β1 siRNA significantly decreased the TGF-β1 mRNA levels in HG-treated GECs and related exosomes (Fig. 6B).

To validate the role of exosomal TGF-β1 mRNA in mediating the exosome-induced podocyte EMT, we incubated podocytes with exosomes silenced for TGF-β1 mRNA and examined the expression of TGF-β1, β-catenin, active β-catenin, Snail, nephrin, ZO-1, WT1, α-SMA, FSP-1, and desmin by Western blotting and immunofluorescence. The results show that when podocytes were co-cultured with exosomes silenced for TGF-β1 mRNA, there was no significant change in the expression of TGF-β1, β-catenin, active β-catenin, Snail, nephrin, ZO-1, WT1, α-SMA, FSP-1, or desmin, indicating that exosomal TGF-β1 mRNA probably mediated elevated TGF-β1 expression levels in podocytes and induced the podocyte EMT (Fig. 6C).

## Discussion

Many studies on diabetes have found that soluble factors that mediate abnormal GEC-podocyte crosstalk may destroy filtration-barrier integrity and eventually lead to albumin leakage. Recently, an increasing body of evidence suggests that exosomes can transfer functional molecules to target cells and serve as mediators of cell-to-cell crosstalk under physiological and pathological conditions. However, in diabetes, whether exosomes can mediate the interaction between GECs and podocytes and participate in the pathogenesis of DN remains unclear. Our research reveals that in HG environments, GECs undergo the EndoMT and produce a notably higher number of exosomes that can trigger the EMT and barrier dysfunction in podocytes. The experimental results illustrate a dramatic induction of TGF-β1 expression and activation of Wnt/β-catenin signaling in the podocytes after treatment with exosomes derived from cells undergoing an EndoMT-like process. We also demonstrate that TGF-β1 mRNA is up-regulated in exosomes from cells undergoing an EndoMT-like process and that exosomes silenced for TGF-β1 mRNA may preserve podocyte phenotypes. In summary, our data indicate that exosomes from HG-treated GECs mediate the EMT and dysfunction of podocytes partly through the transfer of TGF-β1 mRNA.

In many fibrotic diseases, endothelial cells are capable of undergoing the EndoMT to generate myofibroblasts, which lose the endothelial markers CD31 and VE-cadherin, and express high levels of the mesenchymal markers α-SMA and FSP-1. Endothelial cells undergoing the EndoMT can synthesize and secrete fibrogenic/inflammatory cytokines and produce excess ECM in fibrotic lesions, contributing to the development and progression of fibrosis in the lungs, liver, heart, dermis, cornea and kidneys^32^. Previous reports mention that some harmful stimulating factors, such as TGF-β1^33^, CTGF^34^, SiO_2_^35^, serum response factor^36^, hypoxia^37^, can induce an EndoMT-like phenotype. In addition, recent research shows that under pathological conditions, cells undergoing the EndoMT can activate the transdifferentiation of neighboring cells via secretion of profibrotic cytokines in a paracrine-dependent manner^38^. Our experiments revealed that HG can induce the EndoMT in cultured GECs and that the cells undergoing the EndoMT indirectly contribute to renal fibrosis in DN by secreting TGF-β1-containing exosomes that have the capacity to stimulate the EMT and dysfunction of podocytes. These results show the importance of crosstalk between cells undergoing the EndoMT and podocytes for renal fibrosis in DN, suggesting that inhibiting the EndoMT of GECs and reducing the release of TGF-β1-containing exosomes is necessary to halt the progression of renal fibrosis in patients with DN.

In this study, we demonstrated the EMT and dysfunction of podocytes after exposure to the exosomes from cells undergoing the EndoMT for 24 h. Many previous studies have clearly established an intimate linkage of hyperactive TGF-β1 to the podocyte EMT^11^. Therefore, we hypothesized that TGF-β1 would be involved in the exosome-mediated podocyte EMT. As expected, TGF-β1 mRNA and protein expression was markedly up-regulated, and the canonical Wnt/β-catenin signaling was also activated in cultured podocytes after incubation with exosomes from cells undergoing the EndoMT. Because many previous reports have indicated that exosomes can mediate intercellular communication by mRNA transfer, we hypothesized that exosomal TGF-β1 mRNA may be responsible for the EMT and dysfunction of podocytes. Interestingly, TGF-β1 mRNA expression in HG-treated cells undergoing the EndoMT and levels in related exosomes were much higher than those in NG-treated GECs and related exosomes, suggesting that HG can result in the packaging of TGF-β1 mRNA into exosomes, which may then be transferred to podocytes to cause the EMT. However, we cannot rule out the existence of other HG-induced proteins and genetic material in the exosomes secreted by cells undergoing an EndoMT-like process that may potentially play a role in GEC-to-podocyte communication.

In addition to our *in vitro* studies, we have also performed *in vivo* studies by injecting GEC-derived exosomes into C57BL/6 mice via the tail vein 5 times per week for 3 weeks. However, we unexpectedly found that the transport of exosomes secreted from cells undergoing the EndoMT led only to mesangial cells proliferation and the deposition of ECM in the renal mesangial region of mice^26^, but did not cause a significant reduction of nephrin protein levels in podocytes. The reason for this phenomenon may be related to the ultrastructure of the GBM, which is the main filtration barrier against plasma macromolecules based on their size, shape and charge. A normal GBM is a three-dimensional meshwork structure consisting of fine fibrils, which form numerous nearly uniform-sized round pores with diameters of 2.5 ~ 2.8nm^39^. The diameter of the pores is smaller than that of exosomes, preventing GEC-derived exosomes with diameters of 30 ~ 100 nm from reaching the podocytes. However, in diabetes, the meshwork structure of the GBM becomes loosened, and cavities and tunnels with diameters of 10~80 nm or even larger, which do not exist in a normal GBM, are observed in this thickened GBM^40^. Therefore, in diabetes, these enlarged structures allow GEC-derived exosomes to penetrate the GBM to reach podocytes, inducing the EMT and dysfunction.

In summary, our data indicate that HG can induce GEC EndoMT and that cells undergoing the EndoMT can stimulate the EMT and dysfunction of podocytes by releasing exosomes containing TGF-β1. Our findings show the importance of paracrine communication via exosomes between cells undergoing the EndoMT and podoctyes for renal fibrosis in DN. Thus, protecting GECs from the EndoMT and inhibiting the release of exosomes containing TGF-β1 from GECs is necessary to manage renal fibrosis in DN.

## Methods

### Cell culture and *in vitro* studies

The conditionally immortalized mouse podocyte cell line (3111C0001CCC000230) was purchased from National Infrastructure of Cell Line Resource. To propagate cells, podocytes were maintained at 33 °C in low glucose DMEM supplemented with 10% fetal bovine serum (FBS) and interferon-γ. Podocytes were cultured at 37 °C for more than 6 days in the absence of interferon-γ to induce cell differentiation. Mouse primary kidney GECs (C57–6014G, Cell Biologics) were grown in endothelial cell medium supplemented with 5% FBS.

GECs were divided into three groups: normal glucose group (NG; 5.6 mmol/L glucose + 24.5 mmol/L mannitol), high glucose group (HG; 30 mmol/L glucose) and high glucose plus TGF-β1 siRNA group (HG + siRNA) to explore the effect of HG on endothelial phenotype and exosome release from GECs. For the HG + siRNA group, GECs were transiently transfected with TGF-β1 siRNA (Ribobio, China) using ribo*FECT*™ CP Reagent (Ribobio, China) according to the instruction. Exosomes extracted from GEC culture supernatants were also correspondingly divided into three groups: NG-treated GEC-derived exosomes (NG-GEC-Exo), HG-treated GEC-derived exosomes (HG-GEC-Exo) and HG plus TGF-β1 siRNA-treated GEC-derived exosomes (HG + siRNA-GEC-Exo). Podocytes were divided into four groups: podocytes without exosome treatment (Untreated group), podocytes incubated with NG-treated GEC-derived exosomes (NG-GEC-Exo group), podocytes incubated with HG-treated GEC-derived exosomes (HG-GEC-Exo group) and podocytes incubated with HG-treated GEC-derived exosomes silenced for TGF-β1 mRNA (HG + siRNA-GEC-Exo group) to determine the effect of GEC-secreted exosomes on the EMT and dysfunction of podocytes. Previous studies have reported that the ratio of GECs to podocytes is approximately 3:1 in the glomerulus, so this ratio was also in our *in vitro* experiments.

### Exosomes extraction

Differential centrifugation was used to extract exosomes from the serum-free GEC culture supernatants as previously described^41^. In brief, GEC culture supernatants were collected and sequentially centrifugated at 300 × *g* for 10 min, 2,000 × *g* for 15 min, and 10,000 × *g* for 30 min to remove lifted cells, cellular debris and large vesicles. These cleared samples were then subjected to ultracentrifugation at 100,000 × *g* for 70 min twice at 4 °C to pellet the exosomes. The resulting exosomes were resuspended in a small amount of PBS for direct use in subsequent studies.

### Transmission electron microscopy

We used transmission electron microscopy (TEM) to observe the morphology of GEC-derived exosomes by negative staining. Exosome suspensions (2 µl) were distributed dropwise onto a dry slide, and formvar/carbon-coated copper mesh grids were put onto the liquid beads of exosome suspensions to obtain the exosomes for 1 ~ 2 min. Then, the excess suspension on the copper mesh grids was blotted with filter paper. Subsequently, the copper mesh grids were fixed with 2.0% phosphotungstic acid in aqueous suspension for 1 ~ 2 min. Exosome samples were visualized using a Hitachi 7100 transmission electron microscope.

### Western blot analysis

The total protein content from cells or exosomes was extracted, separated by SDS-PAGE and transferred onto polyvinylidene difluoride (PVDF) membranes. The PVDF membranes were blocked with 5% non-fat dry milk in PBS + 0.05% Tween-20 for 1 h and then incubated with indicated primary antibodies overnight at 4 °C, followed by incubation with horseradish peroxidase (HRP)-conjugated secondary antibodies for 2 h at room temperature. The bands were visualized using a gel imaging analysis system (Bio-Rad, USA). The following primary antibodies were used: mouse monoclonal calnexin antibody (1:200; Abcam), mouse monoclonal CD63 antibody (1:1,000; Abcam), mouse monoclonal CD9 antibody (1:500; Abcam), rabbit polyclonal nephrin antibody (1:200; Abcam), mouse monoclonal TGF-β1 antibody (1:1,000; Abcam), rabbit monoclonal (Active) β-catenin antibody (1:1,000; CST), rabbit monoclonal β-catenin antibody (1:1,000; CST), mouse monoclonal Snail antibody (1:1000; CST).

### Quantification of exosome particles

An EXOCET Exosomes Quantitation Kit and FluoroCet Exosome Quantitation Kit (System Biosciences, USA) were used to quantify exosomes according to the manufacturer’s instructions as previously described^26^.

### PKH67 labeling of exosomes and phalloidin staining of podocytes

Firstly, the GEC-derived exosomes were labeled with the green lipophilic fluorescent dye PKH67 (Sigma-Aldrich, St. Louis, MO) according to the instructions. The suspension, containing 2 × 10^9^ exosomes (25 µl), was transferred to a conical-bottom polypropylene tube. A 2× exosome suspension was prepared by adding 1 ml of Diluent C to the exosome suspension, and a 2× dye solution was prepared by adding 4 μl of the PKH67 ethanolic dye solution to 1 ml of Diluent C. Then, 1 ml of 2× exosome suspension was quickly added to 1 ml of 2× dye solution, and the exosomes/dye suspension was incubated for 1 ~ 5 min. Then, 2 ml of 1% BSA was added to bind excess dye.

Podocytes were incubated with PKH67-labeled exosomes for 24 h. The podocytes were fixed with 4% paraformaldehyde for 10 min and then blocked with 1% BSA for 1.5 h at room temperature. Subsequently, podocytes were stained with a 50 μg/ml fluorescent phalloidin conjugate solution (AAT Bioquest, USA) in PBS for 40 min at room temperature. Samples were washed several times in PBS to remove unbound phalloidin conjugate and were observed using a laser scanning confocal microscope (Leica, Germany).

### Immunofluorescence

Podocytes were cultured on the glass slides in 24-well plates and co-cultured with exosomes for 24 h. Then, podocytes were extensively washed 3 times with PBS, fixed in precooling 4% paraformaldehyde for 10 min, permeabilized with 0.1% Triton X-100 for 15 min, and blocked with 10% normal goat serum for 1 h at room temperature. Subsequently, podocytes were incubated with primary antibodies overnight at 4 °C, followed by incubation with secondary antibodies for 2 h at 37 °C. Nuclei were stained with 4′,6-diamidino-2-phenylindole (DAPI). Laser scanning confocal microscope (Leica, Germany) was used to examined the stained podocytes. The following primary antibodies were used: rabbit polyclonal ZO-1 tight junction protein antibody (1:100; Abcam), rabbit polyclonal Wilms’ Tumor protein antibody (1:100; Abcam), rabbit polyclonal α-SMA antibody (1:50; Abcam), rabbit monoclonal S100A4 antibody (1:100; Abcam), mouse monoclonal desmin antibody (1:100; Abcam), rabbit monoclonal β-catenin antibody (1:300; CST), rabbit polyclonal CD31 antibody (1:20; Abcam), and rabbit polyclonal VE-Cadherin antibody (1:200; Abcam).

### Real-time RT-PCR analysis

The total RNA content was extracted from cells and exosomes using TRIzol reagent (Invitrogen) according to the manufacturer’s instructions. Relative expression was calculated using the 2−ΔΔCT method and normalized to the expression of β-actin. For the analysis of TGF-β1 and Wnt 1 expression in cells and exosomes, real-time PCR primers were designed as previously described^42^. TGF-β1 Primer: forward: 5′-GCCCTGGATACCAACTATTGCTTCA-3′, reverse: 5′-CAGAAGTTGGCATGGT-3′. Wnt1 primer: forward: 5′-GCCCTAGCTGCCAACAGTAGT-3′, reverse: 5′-GAAGATGAACGCTGTTTCTCG-3′.

### Albumin influx assay

The albumin influx assay was performed to examine the filtration barrier function of podocyte monolayers as previously described^8, 31^. Transwell chambers with a 0.4-μm pore size (Corning, USA) were used in the albumin influx assay. Podocytes (3 × 10^5^) were seeded on the upper permeable supports and cultured under differentiating conditions for 14 days. The podocytes were serum-starved for 12 h and then incubated with or without GEC (9 × 10^5^)-derived exosomes for 24 h. The upper chamber was then filled with 0.6 ml serum-free DMEM, and the lower chamber was filled with 1 ml serum-free DMEM supplemented with 40 mg/ml of BSA. Total protein concentration in the upper chamber was examined using the bicinchonininc acid (BCA) method at different time points.

### Cell migration assay

To determine the effect of HG on the migration ability of GECs, cell migration assays were conducted as previously described^38^. GECs were grown to confluence in six-well plates, and multiple scratches were made using a 200 μl pipette tip. The GECs were cultured with or without HG (30 mmol/L) for 24 h and then observed and imaged using a Nikon fluorescence microscopy.

### Statistical analysis

SPSS software (IBM, USA) was used to perform statistical analysis. Values are expressed as the mean ± SD. Differences between two groups were assessed using Student’s t-tests, and differences among more than two groups were assessed using an analysis of variance (ANOVA). *p* < 0.05 was defined as statistically significant.

### Data Availability

The datasets generated and analyzed during the current study are available from the corresponding author upon reasonable request.
